# Impact of Vancomycin trough levels monitoring on uncomplicated *methilcillin-resistant Staphylococcus aureus* bacteremia in chronic kidney disease on hemodialysis, retrospective cohort

**DOI:** 10.1186/s12879-024-08984-z

**Published:** 2024-06-25

**Authors:** Julian Felipe Ramirez-Osorio, Juan Esteban Velez-Hernandez, Nathalia Fernandez-Castaño, David Felipe Rojas-Hernandez, Fabian Jaimes

**Affiliations:** 1 Department of Internal Medicine, Hospital Alma Mater de Antioquia, Carrera 77 B # 47 – 113, 050031 Medellín, Antioquia Colombia; 2 University of Antioquia, Medellín, Colombia; 3 IATERIA Journal, Medellín, Colombia

**Keywords:** CKD-HD, Bacteremia, Vancomycin, Trough levels, MRSA

## Abstract

**Background:**

CKD patients on hemodialysis (HD) with *Staphylococcus aureus* (SA) bacteremia present high morbidity, mortality and increased risk of MRSA. Vancomycin is the antibiotic of choice in these cases, it has a narrow therapeutic margin and inadequate dosage generates a risk of toxicity, therefore, the recommendation is to dosage it through serum levels.

**Methods:**

This is a retrospective cohort study in 3 hospitals of third level of complexity in the city of Medellin in which there were differences in the measurement and implementation of vancomycin25 dosage based on trough levels (VL) in patients with chronic kidney disease on hemodialysis (CKD- HD) with uncomplicated bacteremia based infection by *methilcillin-resistant Staphyloccocus aureus* (MRSA). The primary outcome was the composite of hospital mortality, clinical response (fever, hemodynamic instability and altered consciousness), complications associated with bacteremia, or bacteriological response failure (positive cultures at first week follow-up) at 7 days. The composite variables were analyzed individually as secondary outcomes.

**Results:**

The main unadjusted outcome (OR 1.3, CI 0.6 - 2.7) and adjusted for age, Charlson index, loading dose, initial dose, dosing frequency and MIC to vancomycin (OR 1.2, CI 0.5 - 2.7). Regarding adjusted secondary outcomes: clinical response (OR 1.4 CI 0.3 - 5.8), death (OR 1.3 CI 0.3 - 4.6) and complications (OR 0.9, CI 0.37 - 2.2).

**Conclusions:**

We conclude that the measurement of trough levels in patients with HD-CKD does not modify the composite outcome. The main limitation is the sample size and type of study, randomized control trials may be required to confirm the results presented.

## Background

In Colombia, 6% of patients with CKD require renal replacement therapy (RRT) [1]. In patients with CKD-HD, bacteremia due to SA presents high morbidity, mortality and increased risk of MRSA [2]. Local studies have reported a mortality due to SA of 24-30% [3]. In a study conducted at the San Vicente Fundación Hospital of Medellin, SA bacteremias cases were collected retrospectively for 4 years (2012-2016) the results showed 43% of those infected were in RRT [2].

Vancomycin is the drug of choice for the treatment of MRSA bacteremia [2]. This antibiotic has a narrow therapeutic margin, and inadequate dosage poses a risk of toxicity [4] while underdosing predisposes to therapeutic failure and emergence of resistant microorganisms [5]. Its dosage should be based on the serum levels of the antibiotic and the minimum inhibitory concentration (MIC) of the microorganism, specifically for MRSA [6, 7].

Based on current evidence, the recommended PK/PD parameter for Vancomycin in MRSA infections is AUC/MIC with values between 400 to 700 [6]. In patients on hemodialysis, vancomycin trough levels (TL) are used as [8] surrogate marker for AUC/MIC > 400 [6, 7]. Maintaining pre-dialysis concentrations between 15 and 20 mg/L is considered optimal according to the 2020 IDSA guideline [4, 5]; in silico simulations have been conducted evaluating previous dosing models in HD patients [9] demonstrating low probability of attaining AUC/MIC > 400, also the authors developed a model predicting AUC/MIC 400-700 in 90% of patients with a proposed formula for modifying dosages with TL [10]. A retrospective cohort showed a decrease in mortality when target levels were achieved [7]. One study compared two dosing algorithms, both achieving similar TL, but without including clinical outcomes [11]. Despite the lack of clinical outcomes, the guideline by Rybak et al. [5] recommend dosing in CKD-HD patients based on TL.

Although there is a good correlation between the therapeutic levels of vancomycin and its therapeutic efficacy outside the setting of CKD-HD [12] the evidence to support this recommendation in this group of patients is limited [8, 11]. Additionally, this measurement strategy has not yet been compared in the literature with non-measurement of TL. Therefore, it is essential to understand the implications between measuring TL and non measuring TL related to clinical outcomes such as mortality and therapeutic failure. This study compares the results of patients who received dosing according to TL with those who did not, in terms of clinical and microbiological improvement, mortality and reduction of bacteremia related complications.

## Methods

### Study design

This is a retrospective cohort, data was collected from 3 third level hospitals in Medellín-Antioquia (Hospital Pablo Tobón Uribe [*HPTU*], Hospital San Vicente Fundación - Medellín [*HUSVF*], Hospital Alma Mater de Antioquia [*HAMA*]) with data from cultures with *SA* between January 2013 to January 2021 in HUSVF, January 2014 to January 2022 in HAMA and HPTU.

### Participants

#### Inclusion criteria

Patients over 18 years of age with CKD on HD hospitalized for uncomplicated MRSA bacteremia treated with vancomycin. Exclusion criteria were defined as: bacteremia with another associated germ, treatment with antimicrobials different to vancomycin (as adjuvant or definitive therapy) after the diagnosis of MRSA bacteremia, initial cointervention with other antimicrobials effective against MRSA and complicated bacteremia in the first 48 h after the patient's admission. Cohorts of TL dosing and dosing without TL were assigned depending on the monitoring and if required dose modification in each individual patient. Institution #3 mesures TL levels in all patients due to institutional protocol. In institutions #2 and #1 the dosing with TL levels is personalized and contingent upon the individual decisions of treating physicians.

### Variables

The primary outcome was a composite at one-week follow-up, including hospital mortality, clinical response (fever > 38.2 C, hemodynamic instability [SBP < 90 MAP < 60] and altered consciousness [Glasgow < 15]), bacteremia-associated complications (endocarditis, abscesses, discitis, arthritis, empyema, among others) or bacteriological response failure (positive cultures at the end of the first week of follow-up). Secondary outcomes are the individual derivatives of the composite outcome. The quantitative variables used were: weight, TL measurements, cumulative dose, loading dose, initial dose in grams, antibiotic time since symptom onset, treatment duration and Charlson index. These were presented as means and standard deviations. As clarification, institutions do not measure AUC levels in CKD-HD patients. In cases where vancomycin was administered post-hemodialysis, TL levels were assessed prior to the dialysis session.

### Databases

Initial database contained the cultures positive to SA, the data was filtered by MIC greater than or equal to 2 for oxacillin and blood cultures, then the medical records were reviewed individually and included depending on inclusion and exclusion criteria.

### Bias

Measures done to avoid bias included: patients were admitted at the same time of the disease (suspected bacteremia, uncomplicated bacteremia in the first 48 h), knowing the heterogeneity of the sample as it was a cohort study, a multivariate analysis by logistic regression was performed. The assignment of the outcome was based on an exhaustive review of clinical histories by the principal investigators; if there were doubts between the assignment of outcomes, the specific case was discussed between the principal investigators.

### Sample size

Sample size calculation was based in data of Young et al. and Mical et al. [11, 12] both studies in infections caused by MRSA and comparing vancomycin with teicoplanin or trimethoprim sulfamethoxazole. Assuming an outcome frequency of 40% in patients without use of VN, versus 30% in those with VN dosing of the antibiotic, with an exposed/not exposed ratio of 0.5, alpha error of 0.05 and beta error of 0.2; the calculated sample size was 852 patients: 284 with trough levels and 568 with conventional treatment.

### Statistical analysis

Means and medians were calculated for quantitative variables; percentages were calculated for qualitative variables, both for the total cohort and for the cohort dosed by TL and without TL dosing. The unadjusted primary outcome was initially performed by binomial logistic regression and then multivariate adjustment for age, Charlson index, loading dose, initial dose, dosing frequency and MIC to vancomycin. Secondary outcomes were assessed with and without adjustment in a similar manner through binomial logistic regression. The primary database was created in *Microsoft Excel version 16.71*. *Jamovi 2.3.25.0*. was used as statistical program.

## Results

### Patients

**Fig. 1:**
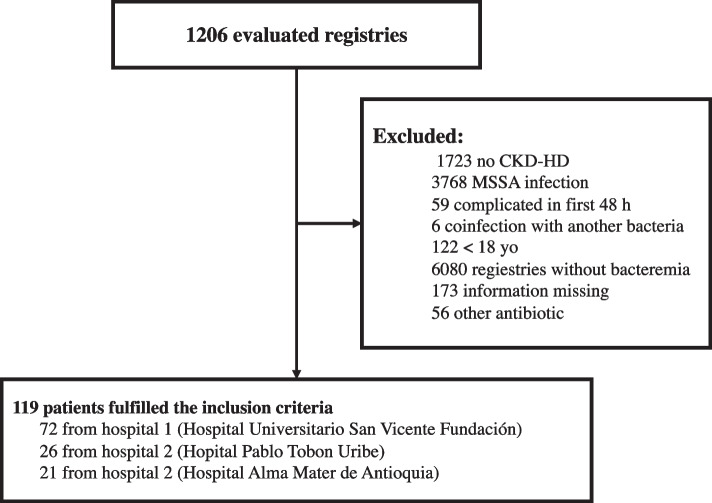
Flowchart illustrating patient selection process

Clinical records of patients with positive cultures for SA were reviewed, being 10580 from hospital one, 391 from hospital two and 1135 from hospital three. The total number of patients included from each hospital was 21, 26 and 72, respectively, for a total of 119 patients. The main cause of exclusion was non-hemodialysis (7423 patients) and *methicillin-sensitive* SA (MSSA) (3768). The remaining variables were infrequent (causes of exclusion are specified in Fig. 1).

### Descriptive data

Majority of patients were from hospital one *n* = 72 (60.5%), 60 men (50%), mean age was 54.13 ± 17.35 years. Of the total number of infections, *n* = 104 (87.4%) were catheter-associated bacteremia, most of them of community origin *n* = 81 (68%). Of the clinical characteristics at admission, the most frequent was tachycardia *n* = 77 (64%). Related to comorbidities, the most frequent was diabetes mellitus (DM) *n* = 59 (49%). Median Charlson index was 5 (RIQ: 3 - 7). In patients with TL measurements, only *n* = 19 (29.2%) were on target levels at the first measurement, most TL were supra-therapeutic *n* = 36 (55.3%). Most frequent dosing interval was every 24 h *n* = 80 (67%). Most received antibiotic co-intervention in the first 48 h *n* = 101 (85%), mainly with piperacillin-tazobactam *n* = 66 (55%). No patient in the cohort had MIC > 1, and most had MIC: 1 *n* = 73 (61%). Of the included patients *n* = 65 (54%) were dosed using at least one TL and *n* = 54 (46%) were dosed without considering TL. Both cohorts are similar in their main characteristics, differences are observed in relation to the collection center as in hospital three all patients were dosed based on TL, while in hospital one the majority (60%) were dosed without TL. There was also a significant difference in onset of symptoms and the beginning of antibiotic therapy, being shorter in the group dosed based on TL. The main missing data in the cohort were follow-up cultures, at 72 h *N* = 39 (32.7%) and at one week *N* = 38 (31.2%) most being after negative cultures at 72 h. Individual patient characteristics are listed in Table 1.

**Table 1:** Sociodemographic, clinical, laboratory, microbiologic and treatment characteristics

**Sociodemographic variables**	**Full cohort *n* = 119 (%)**	**TL dosing *n* = 65 (%)**	**Dosing without TL *n* = 54 (%)**
Hospital 1	72 (60.5)	29 (44.6)	43 (79.6)
Hospital 2	26 (21.8)	15 (23.1)	11 (20.7)
Hospital 3	21 (17.6)	21 (32.3)	0 (0.0)
Age *years* (Mean ± SD)	54.1 ± 17.3	53.2 ± 17.4	55.3 ± 17.4
Weight *kg* (Mean ± DE)	65.4 ± 19.7	66.4 ± 18.3	67.5 ± 16.4
Female	59 (49.6)	37 (56.9)	22 (40.7)
Male	60 (50.4)	28 (43.1)	32 (59.3)
**Clinical variables**			
Tachycardia^a^	77 (64)	41 (63.1)	33 (61.1)
Hypotension^a^	18 (15.1)	9 (13.8)	10 (18.5)
Fever^a^	71 (59.7)	43 (66.1)	28 (51.8)
Glasgow < 15	5 (4.2)	1 (1.5)	4 (7.4)
AMI	16 (13.4)	8 (12.3)	8 (14.8)
CHF	33 (27.7)	20 (30.7)	13 (24.0)
PAD	17 (14.3)	11 (16.9)	6 (11.1)
Stroke / TIA	10 (8.4)	4 (6.1)	6 (11.1)
COPD	11 (9.2)	6 (9.2)	5 (9.2)
Dementia	5 (4.2)	1 (1.5)	4 (7.4)
CTD	15 (12.6)	10 (15.4)	5 (9.2)
PUD	1 (0.8)	1 (1.5)	0 (0.0)
Hepatic disease	5 (4.2)	2 (3.0)	3 (5.5)
DM	59 (49.6)	33 (50.7)	26 (48.1)
Solid tumor	4 (3.3)	0 (0)	4 (7.3)
Leukemia / Lymphoma	6 (5.0)	2 (3.1)	4 (7.4)
AIDS	0 (0.0)	0 (0.0)	0 (0.0)
Charlson index IQR	5 (3 - 7)	5 (3 - 7)	6 (3-7)
Non steroidal immunosupresants	12 (10.8)	8 (12.3)	4 (7.4)
Past medical history of endocarditis	3 (2.5)	1 (1.5)	2 (3.7)
Mitral biologic valve	2 (1.7)	1 (1.5)	1 (1.8)
Catheter associated bacteremia	105 (88.2)	58 (89.2)	47 (87.0)
Community adquired bacteremia	81 (68.0)	47 (72.3)	34 (62.9)
Hospital adquired bacteremia	38 (31.9)	18 (27.7)	20 (37.0)
Catheter lock therapy	3 (2.5)	2 (3.1)	1 (1.8)
Permanent catheter	97 (81.5)	55 (84.6)	42 (77.7)
Femoral catheter	10 (8.4)	5 (7.7)	5 (9.2)
AB timing since symptom onset (hours, mean ± SD)	38.2 ± 38.2	33.2 ± 36.0	36.8 ± 41.1
Catheter retrival since symptom onset (days, mean ± DE)	4.1 ± 3.0	3.6 ± 2.7	4.6 ± 3.3
Consolidation ^b^ AB with V	106 (89.0)	56 (86.1)	50 (92.6)
Daptomycin	9 (7.6)	7 (10.8)	2 (3.7)
AB consolidation with other medication	4 (3.4)	2 (3.1)	2 (3.7)
**Vancomycin**			
TL by por turbidimetry	12 (10.1)	12 (18.5)	N/A
TL by chemiluminescence	30 (25.2)	30 (46.1)	N/A
Unknown method	23 (19.3)	23 (35.4)	N/A
TL 1 (mg/L, media ± DE)	24.7 ± 13.8	24.7 ± 13.8	N/A
% TL < 15 mg/L	10 (8.4)	10 (15.4)	N/A
% TL 15–20 mg/L	19 (15.1)	19 (29.2)	N/A
% TL > 20 mg/L	36 (30.2)	36 (55.3)	N/A
**Microbiológicos**			
MIC < 0.5	44 (37.0)	25 (38.5)	19 (29.2)
MIC 1	73 (61.3)	39 (60.0)	34 (63.0)
**Tratamiento**			
Accumulated dose (g, mean ± SD)	8.6 ± 4.1	8.8 ± 3.9	8.5 ± 7.5
Loading dose (g, mean ± SD)	0.6 ± 0.2	0.5 ± 0.5	0.3 ± 0.5
No loading dose	73 (61.3)	33 (50.8)	40 (74.1)
Initial dose (g, mean ± SD)	0.66 ± 0.24	0.7 ± 0.2	0.6 ± 0.2
Frequency of initial dosage q 12 h	4 (3.4)	4 (6.1)	0 (0.0)
Frequency of initial dosage q24 h	80 (67.2)	34 (52.3)	46 (85.1)
Frequency of initial dosage q 48 h	29 (24.4)	22 (33.8)	7 (12.9)
Frequency of initial dosage q 72 h	4 (3.4)	3 (4.6)	1 (1.8)
AB cointerventions in the first 24–48 h	101 (85.0)	57 (87.7)	44 (81.4)
Didn’t received cointervention	16 (13.4)	8 (12.3)	8 (14.8)
Cointervention with Piperacilina/tazobactam	66 (55.4)	38 (58.4)	28 (51.8)
Cointervention with Cefazolin	7 (5.8)	5 (7.6)	2 (3.7)
Cointervention with other antibiotic	28 (23.5)	14 (21.5)	14 (25.9)
Duration of treatment with V (days, mean ± SD)	16.81 ± 8.46	18.0 ± 9.5	15.4 ± 6.7

*AMI* Acute myocardial infarctation, *CHF* Cardiac heart failure, *PAD* Peripheral artery disease, *TIA* Transient ischemic attack, *COPD* Chronic obstructive pulmonary disease, *CTD* Connective tissue disease, *DM* Diabetes mellitus, *AIDS* Adquired immunodeficiency syndrome, *IQR* Interquartile range, *AB* Antibiotic, *V* Vancomicina, *TL* Throgh level, *SD* Standar deviation

^a^Tachycardia FC > 90. MAP < 60 o SAP < 90. T > 37.8

^b^Patients that finished treatment with vancomycin or change to other AB after day 7

### Outcomes

Primary outcome in the entire cohort occurred *N* = 46 (38%), in patients who were dosed with TL *N* = 27 (41%) and in 19 (35%) of those who were not dosed with TL. The main complication was pulmonary septic emboli with *N* = 20 (16%), followed by hospital mortality with *N* = 14 (11%) and infective endocarditis with 12 (10%). The rest of the outcomes were infrequent and similar between groups as shown in Table 2*.*

**Table 2:** Clinical and microbiologic outcomes

**Outcome variables**	**Full cohorts *n* = 119 (%)**	**TL dosing *n* = 65 (%)**	**Dosing without TL *n* = 54 (%)**
Any complication	36 (30.2)	21 (32.3)	17 (31.5)
Lung septic embolism	20 (16.1)	10 (15.4)	10 (18.5)
Endocarditis	12 (10.1)	8 (12.5)	4 (7.4)
ST abscess	3 (2.5)	3 (4.6)	0 (0.0)
Ab abscess	1 (0.8)	1 (1.5)	0 (0.0)
B abscess	0 (0.00)	0 (0.0)	0 (0.0)
Discitis	2 (1.7)	1 (1.5)	1 (1.8)
Septic arthritis	1 (0.8)	1 (1.5)	0 (0.0)
OM	1 (0.8)	0 (0.0)	1 (1.8)
Empiema	2 (1.7)	0 (0.0)	2 (3.7)
Thromboflebitis	8 (6.7)	3 (4.6)	5 (9.25)
Meningitis	1 (0.8)	1 (1.5)	0 (0.0)
Hepatic abscess	1 (0.8)	1 (1.5)	0 (0.0)
Splenic abscess	1 (0.8)	1 (1.5)	0 (0.0)
Other complications	3 (2.5)	3 (4.6)	0 (0.0)
Possitive cultures at 72 h	30 (25.2)	19 (29.2)	11 (20.4)
Culture was not performed at 72 h	39 (32.7)	19 (29.3)	20 (37.0)
Possitive cultures end of the week	16 (13,4)	10 (15.4)	6 (11.1)
Culture was not performed at end of the week	38 (31.9)	24 (36.9)	22 (40.7)
Inhospital death	14 (11.8)	8 (12.3)	6 (11.1)
Hemodynamic instability*	12 (10.08)	7 (10.7)	5 (9.2)
Primary outcome	46 (38.6)	27 (41.5)	19 (35.18)

*ST* Soft tissue, *Ab* Abdominal, *B* Brain, *OM* Osteomyelitis. *Systolic arterial pressure <90 Median arterial pressure <60

An unadjusted OR of 1.3 (95% CI: 0.6 - 2.7) was found when comparing patients dosed based on TL to those who did not dose based on these levels. After adjusting for the main confounding variables (Charlson index, age, loading dose, initial dose in grams, dosing frequency and MIC to vancomycin), the adjusted OR was found to be 1.2 (95% CI: 0.56- 2.7). While demonstrating a higher proportion of the primary outcome in patients dosed with levels, the association was not statistically significant Table 3.

**Table 3:** Primary and additional outcomes

**Non adjusted outcome**	**OR**	**IC 95%**	***p***
Primary outcome	1.3	(0.6 - 3.9)	0.4
Hemodynamic instability	1.8	(0.3 - 3.9)	0.8
Complications	1.0	(0.5 - 2.2)	0.8
Death	1.1	(0.4 - 3.5)	0.8
**Adjusted otcomes**			
Primary outcome	1.2	(0.3 - 2.7)	0.7
Hemodynamic instability	1.4	(0.3 - 5.8)	0.7
Complications	0.9	(0.3 - 2.2)	0.8
Death	1.1	(0.3 - 4.5)	0.7
**Non adjusted additional outcomes**			
First TL > 15 mg/L	0.6	(0.1 - 2.3)	0.4
Dosage q 12 h	0.6	(0.1 - 4.5)	0.6
Dosage q 24 h	1.1	(0.5 - 2.5)	0.7
Dosage q 72 h	1.6	(0.2 - 11.9)	0.6
**Adjusted additional outcomes**			
First TL > 15 mg/L	0.4	(0.1 - 2.4)	0.3
Dosage q 12 h	0.6	(0.1 - 5.0)	0.6
Dosage q 24 h	0.8	(0.3 - 2.2)	0.9
Dosage q 72 h	1.5	(0.1 - 13.2)	0.6

### Other analysis

#### Secondary outcomes

Clinical instability in the adjusted analysis showed no significant association (OR 1.4, CI 0.3 – 5.8), an similarly, complications (OR 0.9, CI 0.4 - 2.2) and mortality (OR 1.3, CI 0.3-4.4) exhibited no statistical significance. There was a trend towards a higher proportion in the group of patients with VN measurement, except for complications.

As additional analysis, we compared the difference of the primary outcome in patients with TL in therapeutic range (> 15 mg/L) with those who did not achieve these levels (< 15 mg/L) finding (OR 0.5, CI 0.1-2.5). We also calculated the odds for the primary outcome comparing those who dosed every 24 h with respect to those who used another dosing frequency, finding (OR 0.8, CI 0.3-2.2), other supplementary analysis are found in Table 3.

## Discussion

### Main findings

In the present study, no difference was found between the cohort dosed with and without TL, with a trend (without statistical significance) to a greater number of adverse outcomes (microbiological failure, clinical instability, complications and hospital mortality) in patients dosed with TL. Regarding safety, the main adverse effect (acute kidney injury) is not a variable to be considered in these patients.

### Interpretation

There are few studies of MRSA bacteremia and CKD-HD. In the study of Valdés et al., 60% were associated with catheters and 88% of these were due to MRSA [13]. On the other hand, in the study by Cuervo et al. 15.6% of MRSA bacteremias were in HD patients and this subgroup, patients were more comorbid, had a higher MIC of vancomycin (60% had > 1.5 mg/L measured by E-test) and vancomycin was the definitive management in 83% of the cases [14]. In contrast to the above, the present study found that 88% of uncomplicated bacteremias were catheter-associated and 60% of isolates had a MIC of 1 to vancomycin by Vitek method®.

Regarding the dosing of vancomycin in HD, guidelines [5] recommends maintaining trough levels between 15-20 mg/l in order to guarantee efficacy [12]. Rambaran and collaborators in a retrospective cohort of patients with CKD-HD found that mortality was lower with TL between 15 to 29 mg/l OR 0.5 (95% CI 0.63 - 0.9), but did not compare the measurement of TL with not measuring them [8]. The study by Lobna et al. found that there was no difference in the achievement of target levels between measuring VN twice a week vs. measuring it before each HD session, without considering clinically relevant outcomes [11]. In silico studies with Monte Carlo simulations have predicted > 90% probability of target attainment (AUC/MIC 400-700) differentiating regimens according to intradialisis, postdialisis and if the dialysis is done with high flux or low flux membranes, being a start for proposing clinical studies in real world [10]. To our knowledge, this is the first study that compared a dosing group based on TL and another without measuring them, considering therapeutic efficacy in its outcomes.

This study suggests that, due to the erratic elimination of vancomycin in hemodialysis patients, a regular, standard dosing schedule (the most frequent was every 24 h) is similar to level based dosing and suggests that adequate blood levels are achieved with this type of dosing.

Previous studies have shown that achieving TL above 15 is associated with better clinical outcomes, which is confirmed in the additional analysis of the study [5, 11, 12, 15].

### Strengths and limitations

The strengths of this study include the design as a cohort because it provides information on the measurement of TL in CKD-HD and its performance in therapeutic efficacy in real life (without interference from the strict rules that limit the generalization of clinical trials). In addition, another strength is the exhaustive characterization of the population, since it evaluates demographic, clinical, microbiological and treatment variables (that have not been considered in similar studies). With respect to the comparison, to our knowledge this is the only study to date that questions the need for measurement of vancomycin TL in this population, comparing measurement vs. no measurement with respect to clinically relevant outcomes. We also emphasize the need for exploratory analyses for TL > 15 mg/L and depending on the dosage form (12 h, 24 h and 72 h) as it is heterogeneous in this study.

The most common dosing schedule was every 24 h; the dosing recommendation in this population is after hemodialysis so we do not know if the results would be different with a higher proportion of patients dosed every 72 h.

The results of this study should be interpreted in the context of the limitations inherent to its design and the characteristics of the databases collected. Being a retrospective study, it is difficult to control for the risk of unmeasured confounding variables; logistic regression adjustment was performed for the main and secondary outcomes, for variables that could reasonably affect the proportion of the outcome. Also, the dose modification according to TL values was personalized and contingent upon the individual decisions of treating physicians in institution #1 and #2; is worth noting that certain factors influencing these decisions may be challenging to measure comprehensively.

It should be taken into account that the measurement TL could be a factor that increases the risk of bias due to indication (*selection bias*), the patients whom TL measurements are obtained may have factors (whether known or not) that make the clinician more prone to assess the vancomycin levels; for this reason, one of the institutions (hospital #3) was included, since by institutional protocol they measure TL in all patients who are prescribed vancomycin (regardless of their clinical condition). Regarding TL, it is important to highlight that in institution #3, since it is under institutional protocol, there is greater control over pre-analytical variables and staff training for sample collection, which cannot be assured in other institutions.

With respect to the sample size, it was calculated based on a clinical trial and an observational study [16, 17] with a similar composite outcome, the desired number being 852 patients. Due to inherent limitations of the databases, this objective was not achieved; it should be clarified that the search strategy (by means of cultures) reduces the probability of losing patients due to erroneous diagnostic classification.

### Applicability and closure

It should be noted that the focus of this study was on patients with CKD-HD. While this group of patients is particularly vulnerable to infections and RRT-related complications, our findings may not be generalizable to other patient groups. It is important to keep in mind that vancomycin dosing without requiring TL is an important finding, as it reduces the costs and complexity of dose administration; whether there are significant differences in therapeutic effectiveness is to be assessed in prospective randomized clinical trial-type studies.

Given that the MRSA bacteremia is a major public health problem worldwide, we recommend that additional clinical trials be conducted to evaluate the effectiveness and safety of vancomycin dosing according to TL´s compared to no measurement in the ERC-HD population.

## Data Availability

The raw data set was shared with the editors, also the tables, frequencies and ORs calculation from Jamovi *2.3.25.0*. ®. For those interested in obtaining the study´s raw data for academic purporses, please contact the corresponding author, Juan E. Velez-Hernandez at juan.velez30@udea.edu.co.

## Abbreviations

AUC/MIC: Area under de curve to minimum inhibitory concentration
CKD: Chronic kidney disease
DM: Diabetes mellitus
HD: Hemodialysis
HAMA: Hospital Alma Mater de Antioquia
HPTU: Hospital Pablo Tobón Uribe
HUSV: Hospital Universitario San Vicente
MIC: Minimum inhibitory concentration
MRSA: Methilcillin resistant staphylococcus aureus
MSSA: Methicillin succeptible staphylococcus aureus
SA: Staphylococcus aureus
TL: Trough levels
VL: Vancomycin trough levels
